# Effects of a standardized nursing care program on patient outcomes and nursing competence in diabetic patients at a primary hospital

**DOI:** 10.3389/fmed.2026.1649666

**Published:** 2026-02-12

**Authors:** Yi Chen, Meiyuan Dong, Jijuan Liu, Jiujiu He, Hui Liu, Yaner Zhang

**Affiliations:** 1 Nephrology Department, Zhoushan Hospital of Zhejiang Province, Zhoushan, Zhejiang, China; 2 Nursing Department, Zhoushan Hospital of Zhejiang Province, Zhoushan, Zhejiang, China; 3 Gastrointestinal Surgery, Zhoushan Hospital of Zhejiang Province, Zhoushan, Zhejiang, China; 4 Endocrinology Department, Zhoushan Hospital of Zhejiang Province, Zhoushan, Zhejiang, China; 5 Department of Orthopaedics, Zhoushan Hospital of Zhejiang Province, Zhoushan, Zhejiang, China; 6 Infection Management Office, Zhoushan Hospital of Zhejiang Province, Zhoushan, Zhejiang, China

**Keywords:** diabetes mellitus, glycemic control, nursing training, patient satisfaction, standardized homogenized nursing

## Abstract

**Background:**

Diabetes is a major global public health concern. In primary care settings, a significant portion of inpatient diabetes management is delivered by non-endocrinology nurses, yet deficits in their diabetes knowledge can pose risks to patient safety. Therefore, evaluating structured training programs for these nurses is crucial.

**Objectives:**

This study aimed to evaluate the clinical effects of a standardized nursing care program on patient outcomes and nursing competence in diabetic patients at a primary hospital.

**Methods:**

This was a quasi-experimental pre-post controlled study. The control group consisted of 56 nurses and 80 diabetic patients receiving routine care between May 2021 and May 2022. The observation group included the same 56 nurses and a new cohort of 80 diabetic patients after a standardized nursing intervention (June 2022–June 2023). The intervention comprised systematic diabetes nursing training for nurses and the implementation of a standardized care model for patients. Primary outcomes were changes in nurses’ diabetes knowledge and operational scores, and patients’ diabetes knowledge scores, glycemic control, incidence of adverse reactions, and satisfaction. Data were analyzed using Student’s t-test, repeated-measures ANOVA or Chi-square tests.

**Results:**

Compared with the control group, the observation group had higher operational scores and theoretical scores after training (*p* < 0.001). After the intervention, the scores of diabetes knowledge of the patients in the observation group were significantly higher than those in the control group (*p* < 0.001), the levels of fasting blood glucose and glycosylated hemoglobin in the observation group (6.25 ± 0.45 mmol/L and 6.50 ± 0.25%) were lower than those in the control group (6.68 ± 0.58 mmol/L and 6.80 ± 0.20%; *p* < 0.001), the incidence of adverse reactions in the observation group was lower than that in the control group (*p* = 0.034), and the satisfaction rate in the observation group was higher than that in the control group (*p* = 0.003).

**Conclusion:**

The implementation of a standardized nursing care program significantly enhanced the diabetes-related competency of non-endocrinology nurses, leading to improved patient knowledge, better glycemic control, fewer adverse reactions, and higher satisfaction.

## Introduction

In recent years, diabetes has emerged as a formidable global health challenge, with its incidence rising at an alarming rate worldwide. According to the latest data from the International Diabetes Federation (IDF), the global prevalence of diabetes among adults aged 20–79 years reached 10.5% in 2021, and this number is projected to increase to 12.2% by 2045 (1). This escalating trend not only places a substantial burden on individual patients in terms of health and quality of life but also imposes a significant economic strain on healthcare systems globally (2).

The management of diabetes is a complex and multi-faceted process, especially when it comes to in-hospital care. Studies have demonstrated that the proportion of diabetes patients requiring hospitalization varies across different regions. In foreign countries, the hospitalization rate of diabetes patients ranges from 12 to 25% (3). In China, a large-scale epidemiological study revealed that the proportion is 15.1% (4). Moreover, with the rapid advancement of medical science, the field of diabetes care has become increasingly specialized, involving multiple disciplines in the treatment of diabetes and its associated complications (5).

A concerning issue is the high proportion of diabetic patients admitted to non-diabetic specialty hospitals. Statistics indicate that this proportion can be as high as 14.6%, and nearly one-third of the patients in these hospitals have diabetes (6). However, clinical nurses in non - endocrinology departments often lack sufficient knowledge and skills in diabetes management. Common problems such as inconsistent insulin injection techniques, improper handling of hypoglycemia, and incorrect intravenous insulin administration are prevalent. These issues not only reduce the overall quality of medical services but also pose significant safety risks to patients (7).

The current situation highlights a critical research gap. Despite the growing number of diabetes patients in non-endocrinology departments, there is a lack of effective strategies to enhance the diabetes-related knowledge and skills of non-endocrinology nurses. This gap is of great importance because inadequate nursing care can lead to suboptimal glycemic control, which in turn is associated with an increased risk of diabetes-related complications, prolonged hospital stays, and higher healthcare costs (8). Addressing this gap is essential for improving the overall quality of diabetes care in hospitals.

Primary hospitals, in particular, face unique challenges in diabetes management. Due to limited resources and a relatively less specialized workforce, they often struggle to provide high-quality diabetes care (9). Standardized nursing programs can play a crucial role in bridging this gap by offering a structured and consistent approach to diabetes management, regardless of the hospital’s size or resource availability (10).

The concept of hospital standardized management has emerged as a promising solution for improving blood sugar management in non-endocrinology departments. It sets higher requirements and standards for diabetes care in these departments, aiming to ensure that all patients receive high-quality and consistent care (11). Previous studies have shown that standardized management interventions, such as training programs for nurses, can significantly improve nurses’ knowledge and skills in diabetes care, leading to better glycemic control and patient outcomes (12).

Therefore, this study aims to evaluate the effectiveness of a standardized management approach in improving the understanding of diabetes among non-endocrinology nurses and enhancing the quality of nursing management in primary hospitals. By providing comprehensive training on insulin injection, fingertip blood glucose monitoring, and other operations, as well as diabetes-related theoretical knowledge, we hope to improve treatment quality, shorten hospital stays, reduce hospitalization costs, control the occurrence and development of the disease, and ultimately improve the clinical efficacy of patients.

## Methods

### Study design

A quasi-experimental pre-post controlled design was employed to assess the impact of a standardized nursing care program on patient outcomes and nursing competence in diabetic patients at a primary hospital.

This design was chosen because it enables the assessment of changes within the same group of participants over time, which is an effective way to measure the effect of the intervention. The study was divided into two phases: a pre-standardization phase (May 2021–May 2022) and a post-standardization phase (June 2022–June 2023).

### Sampling technique

Cluster sampling was employed in this study. Four departments (neurology, nephrology, gastrointestinal surgery, and orthopedics) were selected as clusters. From these departments, a total of 56 nurses were chosen, and 160 diabetic patients were recruited, with 80 patients in each phase.

### Inclusion and exclusion criteria

Nurses: Inclusion criteria: Registered nurses with a valid practicing license who volunteered to participate in the diabetes specialist care management team were included. Exclusion criteria: Nurses without a nurse qualification certificate were excluded.

Patients: Inclusion criteria: Patients diagnosed with type 1 or type 2 diabetes, with clear consciousness, normal intelligence, and the ability to accurately describe their daily diet and activities were included. Exclusion criteria: Patients with mental, intellectual, or cognitive functional disorders were excluded.

### Sample size justification

The sample size of nurses was determined through power analysis. Assuming a medium-sized effect of the intervention on nurse-related outcomes (e.g., knowledge and skill improvement), with a power of 0.8 and an alpha level of 0.05, a sample size of approximately 50–60 nurses was considered sufficient. Therefore, 56 nurses were selected to ensure adequate statistical power.

For patients, the sample size per phase was calculated via power analysis. Anticipating a moderate effect on patient-related outcomes (e.g., blood glucose control), with a power of 0.8 and an alpha level of 0.05, around 75–85 patients per phase were needed. Considering potential dropouts, the sample size was slightly increased to 80 per phase.

### Ethical considerations

This study was approved by the ethics committee of Zhoushan Hospital of Zhejiang Province on December 29th, 2023 [Approval No. (2023) No. (352)]. Informed consent was obtained from all participants, including nurses and patients, before the start of the study. The participants were informed about the purpose, procedures, potential risks, and benefits of the study, and they had the right to withdraw from the study at any time without any consequences.

### Interventions

#### Control group

Nurses in the control group provided routine diabetes care to patients. This encompassed diet guidance, blood sugar monitoring, and exercise guidance, which are standard components of general diabetes management in a clinical setting.

#### Observation group

The observation group implemented targeted diabetes nursing and scientific management. This was achieved by training diabetes liaison nurses according to standardized nursing care principles. The goal of standardized nursing care was to ensure consistent, high-quality care across different departments within the hospital.

#### Measures to minimize bias

To address potential bias introduced by training nurses in the observation group, several measures were implemented to ensure the fairness and objectivity of the study:

(1) **Training content relevance:** The training content for nurses in the observation group was strictly relevant to diabetes care standardization. It was designed to avoid introducing any factors that would unfairly advantage the observation group patients.
(2) **Patient blinding:** During the study process, patients were blinded as much as possible regarding whether they were in the control or observation group. This was done to reduce potential bias arising from patients’ expectations, which could influence their self-reported outcomes or adherence to treatment.

Specific implementation of standardized nursing service management in the observation group

(1) Organizational structure and leadership

**Leadership setup:** The work was carried out under the direct leadership of the nursing department. A diabetes specialist nurse and an experienced specialist nurse served as deputy leaders. Their responsibilities included training and inspection.

**Liaison nurse selection:** Each department selected a nurse with a strong sense of responsibility as the liaison. These liaison nurses were responsible for safe insulin injection and management within their departments and for timely reporting of any problems.

**Bias reduction in leadership:** Leaders were required to strictly adhere to the standardized training protocols. They were prohibited from providing any additional non-standard care or information to the observation group patients to prevent bias.

(2) Training components, duration, and frequency

(a) Training for liaison nurses

**Training content:** Liaison nurses underwent comprehensive training covering theoretical knowledge and skill operation related to diabetes care. This included safe insulin injection techniques, blood glucose monitoring, management of hypoglycemia, guidance on oral hypoglycemic drugs, patient/family health education, data management, and nursing record management.

**Initial training duration:** The initial training for liaison nurses was conducted over a period of 5 days.

**Refresher training:** Regular refresher training sessions were held on a quarterly basis. This ensured that liaison nurses stayed updated with the latest knowledge and skills in diabetes care.

**Training delivery:** The training was delivered by the diabetes specialist nurse and the experienced specialist nurse who served as deputy leaders, along with external experts in diabetes care if necessary.

**Bias minimization in training:** The training materials and methods were standardized and consistently applied across all liaison nurses. The trainers were trained to avoid any preferential treatment or bias in the training process.

(b) Training for department nurses by liaison nurses

**Training focus:** After mastering the necessary knowledge and skills, liaison nurses conducted training for all nurses in their respective departments. The training focused on the same theoretical knowledge and operational skills as their own training, including standardized procedures for insulin injection, blood glucose monitoring, and patient education.

**Training duration and schedule:** The department-level training sessions were typically conducted over 2 h and were spread out over 2 weeks. This allowed for in-depth learning and practice. These sessions were held as needed, but generally at least once every 2 months to reinforce learning and address any emerging issues.

Training Delivery: The liaison nurses, who had received prior training and had demonstrated proficiency in the relevant knowledge and skills, delivered this training to their department colleagues.

**Bias prevention:** The liaison nurses were instructed to train all department nurses in an objective and consistent manner, without highlighting any potential advantages of the observation group approach beyond the standard content.

(3) Standardized health education

**Problem identification:** For 37 non-endocrinology department health education problems that were not professional, normative, and targeted, a unified health education content for diabetes patients was prepared.

**Training process:** First, systematic training was conducted for liaison nurses on this unified content. Then, after the liaison nurses had mastered it, they trained all nurses in the department. This promoted the realization of standardized management.

**Content basis:** As a unified educational material, the main content of the course was based on the “five carriages,” including diet, exercise, medication, blood glucose monitoring, and education. It also included the prevention of complications and psychological nursing.

**Bias reduction in content development:** The development of the unified health education content was based on widely accepted evidence-based guidelines. The training process ensured that all nurses received the same information without any modifications that could favor the observation group.

(4) Standardized management of blood sugar

(a) Quality supervision

Diabetes specialist nursing organized by the Nursing Department supervised the quality of diabetes management in each department every quarter.

(b) Supervision aspects

Diabetes nursing quality supervision included the following aspects:

Insulin Safety Management: This covered storage requirements, identification of high-risk drugs, and standardized insulin injection.

**Blood glucose meter management:** It included surface cleaning, blood glucose test paper and quality control liquid expiration date labeling, and standardized monitoring of blood glucose.

**Oral hypoglycemic drug management:** Nurses provided guidance on oral hypoglycemic drugs to patients.

**Other aspects:** Patient/family health education, data management, and nursing record management were also included.

(c) Problem feedback and improvement

The problems found in the quality supervision were informed to all departments. Continuous quality improvement was carried out, gradually improving the blood glucose management ability of nurses in all departments and achieving the standardized management of blood glucose of diabetes patients in the whole hospital. To avoid bias in the quality supervision process, the supervisors were trained to be objective and use standardized evaluation criteria. Any identified problems were communicated and addressed in a fair and consistent manner across all departments.

#### Observation indicators

(1) Chinese version of the Michigan Diabetes Knowledge Test (DKT)

**Instrument introduction:** The Chinese version of the Michigan DKT was used to assess the diabetes knowledge of the two groups of patients. The original DKT is a well-established and widely recognized general questionnaire applicable to diabetes patients, consisting of 23 questions.

**Process of adapting the original English version:** Firstly, two bilingual medical professionals with expertise in diabetes knowledge independently translated the original English version of DKT into Chinese. Then, a third bilingual expert compared these two translations, resolved any discrepancies, and generated a unified Chinese draft. Next, two other bilingual personnel who were unaware of the original English version translated the unified Chinese draft back into English. These back-translated English versions were then compared by a native English-speaking diabetes expert with the original English DKT to ensure the accuracy of the translation and the retention of the meaning of each item. A diabetes expert group consisting of endocrinologists, diabetes nurses, and Chinese medical researchers reviewed the Chinese draft. They made necessary revisions to ensure that the content was culturally appropriate and relevant to Chinese diabetes patients. The adapted Chinese version of DKT was pre-tested on 50 diabetes patients in a primary hospital environment similar to this study. Based on the feedback from these patients and the analysis of their responses, the wording of some items was slightly adjusted to improve clarity and understandability.

**Reliability and validity:** In the sample of this study, the Cronbach’s *α* coefficient of the Chinese version of DKT was calculated as 0.89, indicating a high level of internal consistency.

**Structural validity and interpretive threshold:** The structural validity of the Chinese version of DKT was evaluated through exploratory factor analysis. The results showed that the questionnaire could well reflect the underlying structure of diabetic patients’ mastery of diabetes knowledge. Regarding the interpretive threshold, according to the scoring rules of the original DKT, the scores of each question are accumulated, and the total score of the 23-item questionnaire ranges from 0 to 23. The higher the score, the better the mastery of diabetes knowledge. Generally speaking, the scores can be divided into three levels: 0–11 points indicate low-level mastery, 12–17 points indicate medium-level mastery, and 18–23 points indicate high-level mastery.

**Training of evaluators and guarantee of objectivity:** In this study, the distribution and collection of questionnaires were carried out by researchers who had received unified training. The training content included the purpose of the questionnaire, the distribution process, and precautions. During the evaluation process, the researchers strictly followed the scoring rules of the questionnaire to score, avoiding subjective bias and ensuring the objectivity of the evaluation.

(2) Blood glucose-related indicator testing

**Instrument introduction:** On the day of the patient’s admission and 3 months after discharge, fasting blood glucose samples were collected from the patient, and fasting blood glucose and glycated hemoglobin levels were measured. Fasting blood glucose was measured using the Accu-Chek Performa blood glucose meter, and glycated hemoglobin was measured using the Tosoh G8 glycated hemoglobin analyzer.

**Reliability and validity:** Both the blood glucose meter and the glycosylated hemoglobin analyzer have undergone rigorous calibration and quality control, ensuring high accuracy and reliability. The test results of the blood glucose meter are highly consistent with those of the large-scale biochemical analyzer in the laboratory, with a correlation coefficient reaching 0.95; the test results of the glycosylated hemoglobin analyzer also comply with international standards, with a coefficient of variation (CV) less than 2%.

**Structural validity and interpretation threshold:** Fasting blood glucose and glycosylated hemoglobin are important indicators reflecting the blood glucose control status of diabetic patients, and they have clear structural validity. The normal range of fasting blood glucose is generally 3.9–6.1 mmol/L, and a level greater than 7.0 mmol/L may suggest a diagnosis of diabetes; the normal range of glycosylated hemoglobin is 4–6%, and a level greater than 6.5% may suggest a diagnosis of diabetes. In this study, by comparing the fasting blood glucose and glycosylated hemoglobin levels of patients at different time points, the effect of blood glucose control was evaluated.

**Training of assessors and guarantee of objectivity:** The nurses who collect blood samples have all received professional training, are familiar with the blood collection process and precautions, and can accurately collect blood samples. The testing personnel have also undergone strict training, are proficient in the operation methods of blood glucose meters and glycosylated hemoglobin analyzers, and conduct tests in accordance with the operating procedures to ensure the objectivity and accuracy of the test results. At the same time, the instruments are regularly calibrated and maintained to ensure their normal operation.

(3) Evaluation of adverse reactions

**Instrument introduction:** The adverse reactions that occurred in the two groups of patients were compared, including injection site complications, reuse of needles, skin leakage, and improper disposal of discarded needles. The standardized tools were used for assessment, such as using a standardized grading scale to record injection site complications.

**Reliability and validity:** The standardized grading scale has been verified by experts through pre-tests and has high reliability and validity. The content validity index (CVI) of the scale reaches 0.90, and the Cronbach’s *α* coefficient is 0.88, indicating that the scale can accurately and reliably assess the severity of injection site complications.

**Structural validity and interpretation threshold:** The standardized grading scale classifies injection site complications based on clinical manifestations and severity, demonstrating clear structural validity. For instance, redness and swelling are categorized as mild (local redness and swelling diameter < 2 cm), moderate (2–5 cm), or severe (> 5 cm or accompanied by additional symptoms such as fever, purulent exudate). Through this grading method, the severity of injection site complications in patients can be clearly evaluated.

**Training of assessors and guarantee of objectivity:** A team consisting of three independent nurses who have received training and are not involved in the implementation of nursing interventions is responsible for assessing these adverse reactions. Before the assessment, all assessors undergo unified training, which includes the definition of adverse reactions, grading standards, assessment methods, etc. After the training, assessors are evaluated to ensure they can accurately and objectively conduct the assessment. During the assessment process, assessors strictly record according to the standardized grading scale, avoiding subjective judgment, and ensuring the objectivity of the assessment.

(4) Patient satisfaction assessment

**Instrument introduction:** The patient satisfaction survey questionnaire developed by the hospital was used to assess patient satisfaction. This questionnaire was specifically designed to capture various aspects of the diabetes patient’s care experience in the grassroots hospital environment, including communication quality, the effectiveness of nursing services, and the overall nursing environment.

**Reliability and validity:** Before using this research, the questionnaire was pre-tested on a sample of 30 diabetes patients similar to the research population. The Cronbach’s *α* coefficient was calculated to evaluate the internal consistency of the questionnaire, and the result was 0.85, indicating a good internal consistency level. Additionally, factor analysis was conducted to explore the potential structure of the questionnaire items, further supporting the effectiveness of this tool in measuring patients’ satisfaction with nursing services.

**Structural validity and interpretation threshold:** The results of the factor analysis showed that the questionnaire had good structural validity, accurately reflecting the underlying structure of patients’ satisfaction with nursing services. Each item in the questionnaire has a score range of 0–10, where 0 represents the lowest satisfaction and 10 represents the highest satisfaction. By adding up the scores of all items to calculate the total satisfaction score of each patient, the higher the score, the more satisfied the patient is with the received nursing services. Generally, the total score can be divided into three grades: 0–50 is unsatisfactory, 51–75 is basic satisfaction, and 76–100 is satisfaction.

**Training of assessors and objectivity assurance:** The distribution and collection of the questionnaire were completed by trained researchers. The training content included the purpose of the questionnaire, distribution process, instructions, etc. When patients filled out the questionnaire, researchers provided unified guidance to avoid leading language, ensuring that patients could fill out the questionnaire based on their true feelings. At the same time, the collected questionnaires were strictly reviewed to eliminate invalid ones, ensuring the objectivity of the assessment results.

(5) Nurse operation and theory assessment

**Instrument introduction:** After implementing the nursing measures, the operation scores and theory scores of the nurses in both groups were evaluated. The operation scores were based on the unified operation scoring standard of the hospital, and the theory scores were assessed through the knowledge mastery assessment form.

**Reliability and validity:** The unified operation scoring standard and the knowledge mastery assessment form of the hospital have been subject to expert review and multiple revisions, and have high reliability and validity. The Cronbach’s *α* coefficient of the operation scoring standard is 0.92, and the Cronbach’s α coefficient of the knowledge mastery assessment form is 0.90, indicating that both can accurately and reliably assess the operational skills and theoretical knowledge level of nurses.

**Structural validity and interpretation threshold:** The operation scoring standard and the knowledge mastery assessment form are designed based on the actual requirements of nursing work and the professional knowledge of diabetes care, and have clear structural validity. The total score of the operation scoring standard is 100 points, and it is scored based on aspects such as the standardization, proficiency, and aseptic concept of the operation. Scores of 80 points and above are considered excellent, 60–79 points are considered qualified, and scores below 60 points are considered unqualified. The knowledge mastery assessment form includes five items: hypoglycemia, insulin injection, oral medication, diet, and blood glucose testing, with a total score of 100 points. Scores are based on the nurse’s mastery of each item, with 80 points and above considered good mastery, 60–79 points considered basic mastery, and below 60 points considered unmastered.

**Training of assessment personnel and objective guarantee:** The assessment personnel are composed of nursing experts with rich clinical and teaching experience. Before the assessment, they receive unified training to familiarize themselves with the content and scoring methods of the operation scoring standard and the knowledge mastery assessment form. During the assessment, the assessment personnel score strictly according to the standards, avoiding subjective factors, and ensuring the objectivity and fairness of the assessment results.

(6) Nurse knowledge level assessment

**Instrument introduction:** Based on the unified diabetes specialty nursing quality assessment form of the hospital, and through the knowledge mastery assessment form formulated by the hospital, the nurse’s knowledge level is evaluated. This assessment form includes five items: hypoglycemia, insulin injection, oral medication, diet, and blood glucose testing, with a total score of 100 points. The operation score is calculated according to the unified operation scoring standard of the hospital, with a total score of 100 points as well.

**Reliability and validity:** The knowledge mastery assessment form and the operation scoring standard have undergone strict formulation and review processes, and have high reliability and validity. The Cronbach’s *α* coefficient of the knowledge mastery assessment form is 0.91, and the Cronbach’s α coefficient of the operation scoring standard is 0.93, indicating that both can accurately and reliably assess the nurse’s knowledge level and operational skills.

**Structural validity and interpretation threshold:** The knowledge mastery assessment form and the operation scoring standard are designed based on the professional knowledge and skills requirements of diabetes care, and have clear structural validity. The total score interpretation of the knowledge mastery assessment form is as described in (5) above. The operation scoring standard also has a total score of 100 points, and is scored based on aspects such as the accuracy, standardization, and proficiency of the operation, with the grading levels also consistent with those in (5) above.

**Training of assessment personnel and objectivity assurance:** The assessment work is handled by a specialized nursing quality assessment team, and the team members are familiar with the assessment standards and procedures. Before the assessment, a unified training is conducted for the assessment team, emphasizing the objectivity and fairness of the assessment, and avoiding subjective biases. During the assessment process, scoring is carried out strictly according to the assessment standards to ensure the accuracy and reliability of the assessment results. At the same time, the assessment team is regularly evaluated and feedback is provided to continuously improve the assessment quality.

### Statistical analysis

The statistical analyses were conducted using SPSS 17.0 software. Continuous variables are presented as mean ± standard deviation (SD), while categorical variables are reported as frequencies (n) and percentages (%). Prior to conducting any inferential statistical tests, we assessed the normality of the continuous variables using the Shapiro–Wilk test. When the Shapiro–Wilk test indicated that the continuous variables followed a normal distribution, we performed a Student’s t-test to compare the means between two independent groups. For continuous variables that did not follow a normal distribution, we opted for the Mann–Whitney U test. The Chi-square test was used to examine the associations between categorical variables. Repeated-measures ANOVA was performed to identify specific time points at which significant differences occurred in the continuous variables measured over time. Effect size was displayed as 95% confidence interval (95% CI) when applicable. We used multiple imputation to handle missing data. Multiple imputation is a statistical technique that creates several different imputed datasets by filling in the missing values with plausible values based on the observed data. The statistical analyses were then performed on each of the imputed datasets, and the results were combined using Rubin’s rules to obtain overall estimates and standard errors. This approach helps to reduce the bias that can be introduced by simply ignoring missing data or using simple imputation methods such as mean imputation. A *p*-value of less than 0.05 was considered statistically significant throughout the study.

## Results

Comparison of baseline characteristics of nurses and patients between the two groups.

The baseline characteristics of nurses were presented in Table 1.

**Table 1:** Baseline characteristics of nurses.

Item	Numbers or percentage
Gender	
Male	10 (17.86)
Female	46 (82.14)
Age (years)	
Years of working (years)	4.52 ± 0.58
Patient distribution across wards	
Neurology	14 (25.00)
Nephrology	15 (26.79)
Gastrointestinal surgery	13 (23.21)
Orthopedics	14 (25.00)

As shown in Table 2, there were no statistically significant differences in the baseline characteristics of patients between the two groups (*p* > 0.05).

**Table 2:** Baseline characteristics of patients between the two groups.

Items	Control group (*n* = 80)	Observation group (*n* = 80)	*X^2^/t*	*p*
Gender			0.025	0.874
Male	38 (47.50)	37 (46.25)		
Female	42 (52.50)	43 (53.75)		
Age (years)	56.36 ± 11.12	56.43 ± 11.26	0.039	0.968
BMI	28.73 ± 3.25	28.78 ± 3.34	0.096	0.923
Type of diabetes			0.105	0.745
Type 1 diabetes	50 (62.50)	48 (60.00)		
Type 2 diabetes	30 (37.50)	32 (40.00)		
Duration of diabetes (years)	8.90 ± 7.41	8.85 ± 7.52	0.042	0.966
Patient distribution across wards			0.104	0.991
Neurology	25 (31.25)	26 (32.50)		
Nephrology	20 (25.00)	19 (23.75)		
Gastrointestinal surgery	20 (25.00)	21 (26.25)		
Orthopedics	15 (18.75)	14 (17.50)		

### Comparison of diabetic knowledge between the two groups

**Figure 1:**
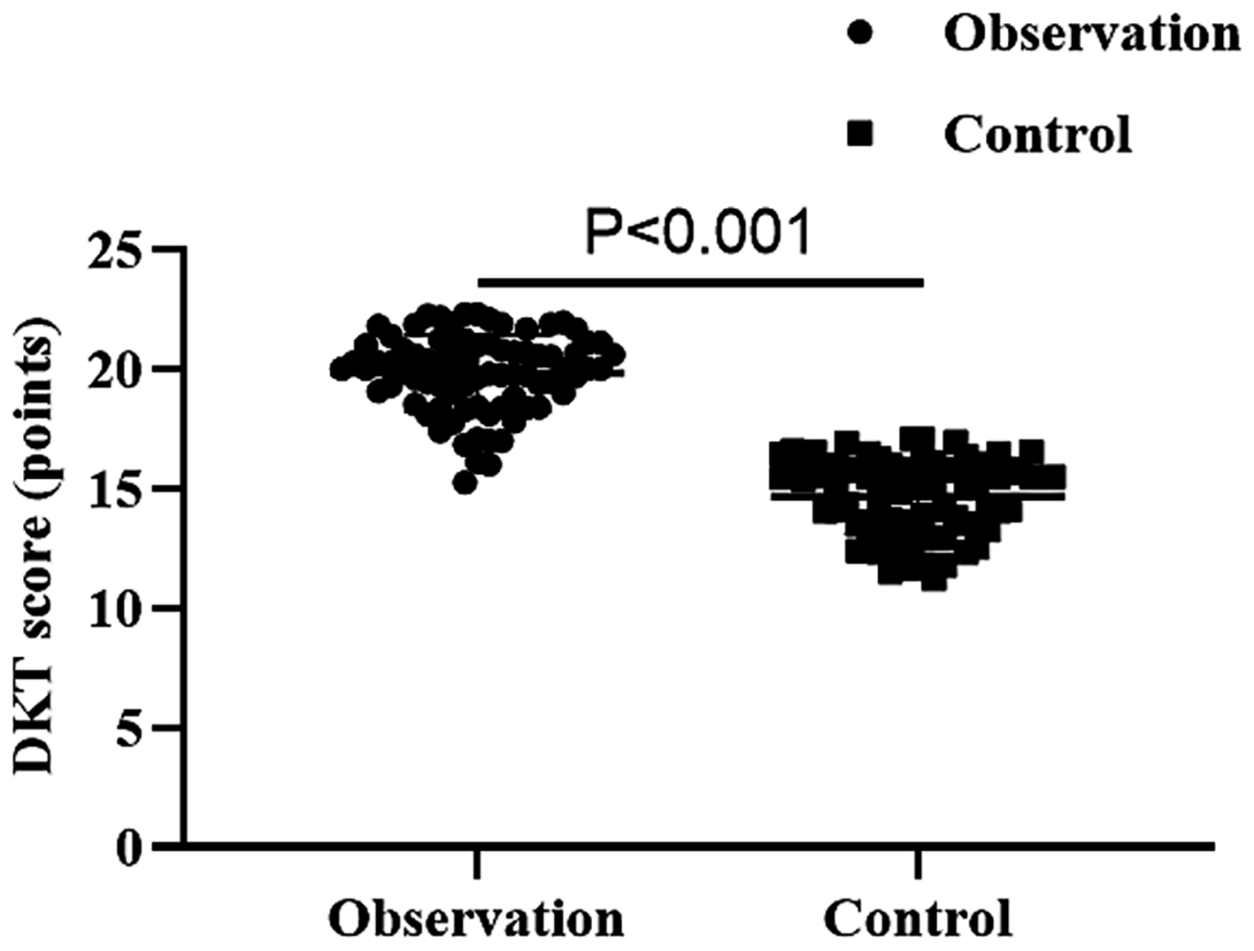
Comparison of diabetic knowledge cognition between the two groups.

As shown in Figure 1, the DKT score of the observation group was higher than that of the control group (*p* < 0.001, 95% CI: −5.666--4.678).

### Comparison of fasting blood glucose and glycated hemoglobin between the two groups

**Figure 2:**
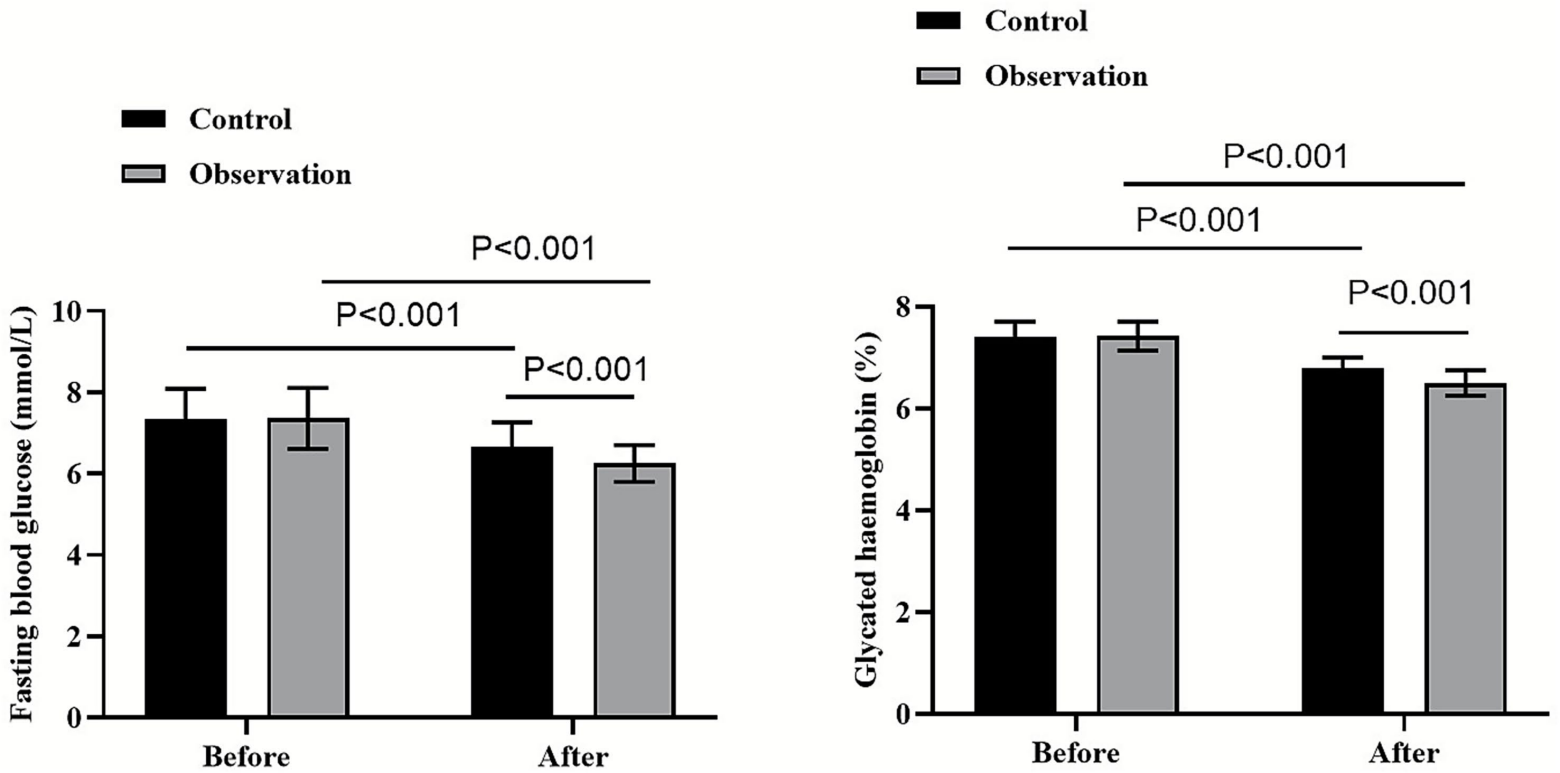
Comparison of fasting blood glucose and glycated hemoglobin between the two groups.

On the day of the patient’s admission, no significant differences were found between the two groups for both fasting blood glucose and glycated hemoglobin (*p* > 0.05). Three months after patient discharge, the levels of fasting blood glucose and glycated hemoglobin in both groups were lower than those on the day of the patient’s admission (*p* < 0.001, 95% CI: 0.068–0.351; *p* < 0.001, 95% CI: 0.082–0.197). More importantly, the levels of fasting blood glucose and glycated hemoglobin in the observation group (6.25 ± 0.45 mmol/L and 6.50 ± 0.25%) were lower than those in the control group (6.68 ± 0.58 mmol/L and 6.80 ± 0.20%) 3 months after patient discharge (*p* < 0.001, 95% CI: 0.748–1.031; *p* < 0.001, 95% CI: 0.702–0.817; Figure 2).

### Occurrence of adverse reactions between the two groups

In the control group, there were 23 cases of complications of injection site, 16 cases of reuse of needle, 14 cases of needle/skin leakage and 14 cases disposal of syringe needles. In the observation group, there were 10 cases of complications of injection site, 6 cases of reuse of needle, 5 cases of needle/skin leakage and 5 cases disposal of syringe needles. Compared with the control group, the occurrence of adverse reactions including complications of injection site, reuse of needle, needle/skin leakage and disposal of syringe needles in the observation group was significantly lower (*p* = 0.011, *p* = 0.021, *p* = 0.027, *p* = 0.027; Table 3).

**Table 3:** Occurrence of adverse reactions in both groups.

Groups	N	Complications of injection site	Reuse of needle	Needle/Skin leakage	Disposal of discarded syringe needles
Control group	80	23 (28.75)	16 (20.00)	14 (17.50)	14 (17.50)
Observation group	80	10 (12.50)	6 (8.0)	5 (6.25)	5 (6.25)
X^2^		6.451	5.270	4.837	4.837
*p*		0.011	0.021	0.027	0.027

### Satisfaction of patients between the two groups

**Figure 3:**
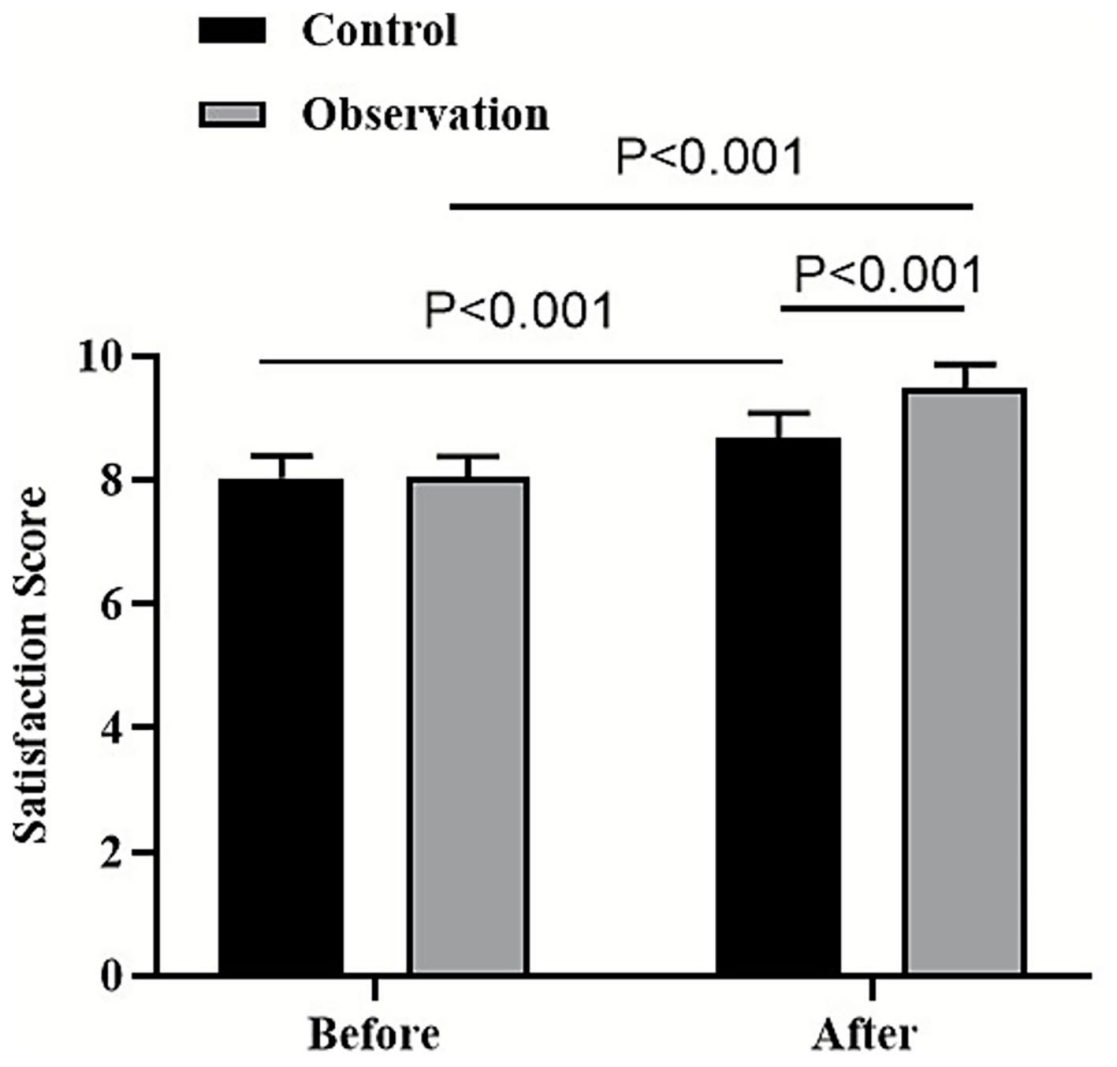
Satisfaction of patients between the two groups.

On the day of the patient’s admission, no significant differences were found in the satisfaction scores of patients between the two groups (*p* > 0.05). Three months after patient discharge, the satisfaction scores of patients in both groups were higher than those on the day of the patient’s admission (*p* < 0.001, 95% CI: −1.111–−0.949). More importantly, the satisfaction scores of patients in the observation group were higher than those in the control group (*p* < 0.05, 95% CI: −0.500–−0.339; Figure 3).

### Assessment of operational scores and theoretical scores between the two groups

**Figure 4:**
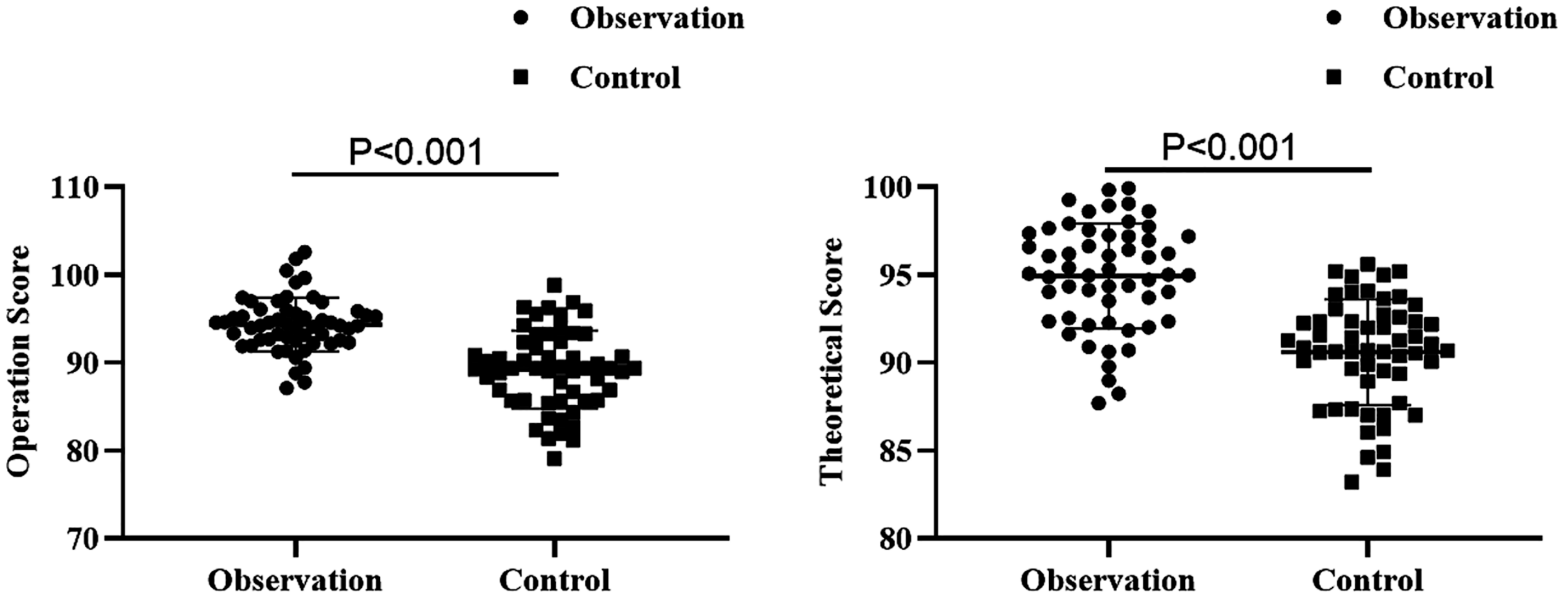
Assessment of operational scores and theoretical scores between the two groups.

Compared with the control group, the observation group had higher operational scores and theoretical scores after nursing (*p* < 0.001, 95% CI: −6.225–−3.666; *p* < 0.001, 95% CI: −5.456–−3.211; Figure 4).

### Knowledge mastery of nurses between the two groups

The knowledge scores of hypoglycemia, insulin injection, oral medicine, diet and blood glucose detection in the observation group were higher than those in the control group (*p* < 0.001, 95% CI: −8.857–−5.863; *p* < 0.001, 95% CI: −9.145–−5.675; *p* < 0.001, 95% CI: −10.26–−6.565; *p* < 0.001, 95% CI: −8.364–−4.996; Table 4).

**Table 4:** Knowledge mastery of nurses in both groups.

Groups	Cases	Hypoglycemic knowledge score	Insulin injection knowledge score	Oral medicine knowledge score	Dietary knowledge score	Blood glucose detection knowledge score
Observation group	56	93.63 ± 3.62	94.17 ± 4.17	92.43 ± 2.15	93.52 ± 3.24	92.95 ± 2.71
Control group	56	86.27 ± 4.34	86.76 ± 5.05	84.58 ± 6.22	85.11 ± 6.14	86.27 ± 5.72
t		9.746	8.467	8.926	9.065	7.898
*p*		<0.001	<0.001	<0.001	<0.001	<0.001
95% CI		−8.857--5.863	−9.145--5.675	−9.605--6.095	−10.26--6.565	−8.364--4.996

## Discussion

The primary objectives of this study were to evaluate the effectiveness of a standardized nursing care program in improving blood sugar levels, reducing adverse reactions, enhancing patient satisfaction, and increasing nurses’ knowledge and skills in diabetes management among non-endocrinology diabetes patients at a primary hospital.

Our results showed that 3 months after patient discharge, the levels of fasting blood glucose and glycated hemoglobin in the observation group (6.25 ± 0.45 mmol/L and 6.50 ± 0.25%) were lower than those in the control group (6.68 ± 0.58 mmol/L and 6.80 ± 0.20%), indicating that the standardized nursing care program can effectively improve blood sugar levels in diabetic patients at a primary hospital. This finding is in line with several international studies. For instance, a study by Valladolid et al. demonstrated that standardized nursing interventions, which included regular patient education and follow-up, led to a significant reduction in both fasting blood glucose and glycated hemoglobin levels (13). However, our study goes a step further by providing a more detailed and structured nursing program that encompasses not only patient education but also standardized operational procedures for nurses. This comprehensive approach may explain the more pronounced improvements in blood sugar levels observed in our study compared to some previous ones.

Compared with the control group, the observation group had a significantly lower occurrence of adverse reactions and a higher satisfaction rate. This is consistent with research by Muhoma et al. (14), which found that standardized nursing care, with its emphasis on consistent and high-quality care delivery, can reduce the risk of complications and improve patient satisfaction. In contrast to some international studies that mainly focused on large-scale tertiary hospitals, our study in a primary hospital setting highlights the feasibility and effectiveness of standardized nursing in a resource-limited environment. The higher satisfaction rate in our study may be attributed to the personalized attention and continuous support provided by the standardized nursing program, which addressed patients’ specific needs and concerns more effectively.

Our study demonstrated that nurses in the observation group had higher operational scores, theoretical scores, and knowledge scores in various aspects of diabetes management, such as hypoglycemia, insulin injection, oral medicine, diet, and blood glucose detection. This is similar to the findings of Şimşek et al. who reported that standardized training programs for nurses can significantly improve their knowledge, skills, attitudes, and competence levels (15). However, our study has the advantage of a more in-depth assessment of nurses’ knowledge across multiple domains, providing a more comprehensive picture of the impact of the standardized nursing care program. The structured training and regular supervision in our program may have contributed to the more significant improvements in nurses’ performance compared to some previous studies.

### Theoretical, practical, and clinical implications

**Theoretical implications**: Our study provides empirical evidence that supports the theoretical framework of standardized nursing in diabetes management. It validates the concept that a structured and unified approach to training and care delivery can lead to improved outcomes. This contributes to the existing body of knowledge in the field of diabetes nursing, suggesting that standardized nursing should be an integral part of diabetes care theory.

**Practical implications**: From a practical perspective, our findings have significant implications for hospital administrators and nursing managers. The implementation of standardized nursing services can enhance the overall quality of diabetes care in non-endocrinology departments. It can help in building a more cohesive and knowledgeable nursing workforce, reducing the risk of errors in blood glucose monitoring and insulin injection, and improving patient satisfaction. This can ultimately lead to better patient outcomes and a more efficient use of healthcare resources.

**Clinical implications**: Clinically, standardized nursing can ensure that patients receive consistent and high-quality care regardless of the department they are in. It can help in the early identification and management of diabetes-related complications, as nurses with standardized training are more likely to have a comprehensive understanding of the disease and its management. This can lead to better blood glucose control, reduced mortality rates, and an improved quality of life for diabetes patients.

### Limitations

This study has several limitations. Firstly, the small sample size and single-center design limit the generalizability of our findings to broader populations and settings. The results may not be applicable to hospitals with different patient demographics, resource levels, or organizational cultures. Secondly, the non-randomized comparison design introduces the potential for selection bias, as participants were not randomly assigned to the intervention and control groups. This may have influenced the results, as the two groups may have had different baseline characteristics that were not fully accounted for. Thirdly, observer bias may have influenced the assessment of outcomes, as the researchers were aware of the participants’ group assignments. This could have led to an over- or under-estimation of the effects of the standardized nursing intervention.

To address the limitations of this study, future research should aim to employ larger sample sizes and multi-center designs. This would increase the external validity of the findings and make them more applicable to a wider range of healthcare settings. Randomized controlled trials should be conducted to minimize selection bias and ensure that the results are due to the intervention rather than other factors. Blinded outcome assessments should also be implemented to reduce observer bias and provide more objective and reliable results. Additionally, future studies could explore the long-term effects of standardized nursing on diabetes patients’ health outcomes, such as the incidence of complications and quality of life over an extended period. They could also investigate the cost-effectiveness of standardized nursing services to provide more comprehensive evidence for decision - making in healthcare management.

## Conclusion

Our study demonstrates that the standardized nursing care program for non - endocrinology diabetes patients in a primary hospital effectively achieves its set objectives. It successfully improves patients’ blood sugar levels, reduces adverse reactions, enhances patient satisfaction, and boosts nurses’ knowledge and skills in diabetes management. However, it is important to acknowledge that our interpretation of these results should take into account the limitations of our study, including the relatively small sample size, the non-randomized study design, and the fact that it was conducted in a single center, which may restrict the generalizability of our findings. Despite these limitations, implementing such a standardized nursing program in primary hospitals can be a practical and beneficial strategy to standardize care quality, minimize safety risks, and offer more patient-centered diabetes management. It provides a replicable model for similar healthcare settings to enhance diabetes care outcomes. Looking ahead, future research should focus on conducting multi-center trials with larger sample sizes and randomized designs to further validate the effectiveness of the standardized nursing care program. Additionally, long-term follow-up studies are needed to assess the sustainability of the program’s benefits over an extended period. These future directions will help to build a more robust evidence base for the widespread adoption of standardized nursing in diabetes care.

## Data Availability

The datasets presented in this study can be found in online repositories. The names of the repository/repositories and accession number(s) can be found in the article/supplementary material.
